# Single-Cell RNA Sequencing Elucidates the Structure and Organization of Microbial Communities

**DOI:** 10.3389/fmicb.2021.713128

**Published:** 2021-07-21

**Authors:** Melanie A. Brennan, Adam Z. Rosenthal

**Affiliations:** IFF, Health & Biosciences, Research & Development, Wilmington, DE, United States

**Keywords:** heterogeneity, cell states, single-cell RNA sequencing, physiology, bet hedging

## Abstract

Clonal bacterial populations exhibit various forms of heterogeneity, including co-occurrence of cells with different morphological traits, biochemical properties, and gene expression profiles. This heterogeneity is prevalent in a variety of environments. For example, the productivity of large-scale industrial fermentations and virulence of infectious diseases are shaped by cell population heterogeneity and have a direct impact on human life. Due to the need and importance to better understand this heterogeneity, multiple methods of examining single-cell heterogeneity have been developed. Traditionally, fluorescent reporters or probes are used to examine a specific gene of interest, providing a useful but inherently biased approach. In contrast, single-cell RNA sequencing (scRNA-seq) is an agnostic approach to examine heterogeneity and has been successfully applied to eukaryotic cells. Unfortunately, current extensively utilized methods of eukaryotic scRNA-seq present difficulties when applied to bacteria. Specifically, bacteria have a cell wall which makes eukaryotic lysis methods incompatible, bacterial mRNA has a shorter half-life and lower copy numbers, and isolating an individual bacterial species from a mixed community is difficult. Recent work has demonstrated that these technical hurdles can be overcome, providing valuable insight into factors influencing microbial heterogeneity. This perspective describes the emerging microbial scRNA-seq toolkit. We outline the benefit of these new tools in elucidating numerous scientific questions in microbiological studies and offer insight about the possible rules that govern the segregation of traits in individual microbial cells.

## Introduction

### The Value of Using Microbial Single-Cell RNA Sequencing to Study Phenotypic Heterogeneity in Bacteria

Although bacteria grown from a single cell or colony are often treated as uniform, heterogeneity in clonal bacterial populations has been observed since the dawn of bacteriology. Koch and Cohn independently discovered sporulating subpopulations of cells via microscopy in 1876, and Roland Thaxter reported that morphologically distinct cells form intricate structures in isolated cultures of myxobacteria in 1892 (Thaxter, 1892). Despite these early discoveries, the function and interactions of different cell-types within clonal microbial communities are only now beginning to be resolved due in part to the immense technical challenges of assaying individual cells. With the aid of fluorescent protein reporters and fluorescence in-situ techniques pioneered in the early 1990s, several interesting observations about the activity and role of cellular heterogeneity in clonal bacterial communities have been made [for review see (Ackermann, 2015; Imdahl and Saliba, 2020)]. Heterogeneity has been observed when bacteria were cultured in both colonies or biofilms and in well-mixed cell suspensions. In spatially structured environments, heterogeneity is environmentally organized and different cells are exposed to unique microenvironments, each with specific challenges and opportunities. Within physically homogeneous conditions, such as well-shaken liquid cultures used in laboratory work, one ecological advantage to cellular heterogeneity is a strategy known as bet hedging in which communities benefit by partaking in different strategies in anticipation of environmental challenges. For instance, while a fraction of a population continues vegetative growth in the face of environmental hardships (such as low nutrient availability or extreme temperatures) in many bacterial species, the risk of deteriorating conditions is hedged by a distinct population of cells that undergo sporulation. In addition to bet hedging, there have been numerous reports about a strategy in which bacteria “divide-labor” in order to more efficiently produce common goods (Lynn and De Leenheer, 2019), and there are examples in which bacterial cell-fractions (subpopulations) emerge to metabolize toxic compounds that accumulate in the culture (Rosenthal et al., 2018).

Despite advancements aided by fluorescence microscopy, studies that rely on fluorescent measurements in single cells are not truly high-throughput and typically require a hypothesis-driven approach that relies on tedious construction of a small number of reporters to answer a specific set of questions. Even in the cases in which genome-wide fluorescent reporter strains exist (for example Zaslaver et al., 2006), measurements are limited in the number of genes that can be multiplexed in individual cells, making it difficult to directly compare the expression of hundreds of individual genes with each other inside a single cell.

The emergence of single-cell RNA sequencing (scRNA-seq) techniques make it now possible to observe transcriptome-wide heterogeneity agnostically, without any preconceived bias introduced in studies when choosing a limited number of genes studied simultaneously. Mammalian scRNA-seq techniques, such as InDrop (Klein et al., 2015), DropSeq (Macosko et al., 2015), and split-pool (Cao et al., 2017; Rosenberg et al., 2018), resulted in discoveries of new cell types and atlases quickly and affordably. In addition to characterizing the heterogeneity landscape, analysis programs, such as Monocle3 (Trapnell et al., 2014), can project cell trajectories over pseudotime, uncovering the underlying regulatory programs that give rise to different cell fates. Spatial transcriptomic tools including seqFISH, MERFISH, and others (Lubeck et al., 2014; Wang et al., 2018) combine single-cell transcriptomics with spatial mapping of each cell. This information allows for the exploration of many scientific questions, but these techniques are only now being applied to bacterial systems.

## Discussion

### Difficulties in Applying Eukaryotic Techniques to Bacteria

**Figure 1:**
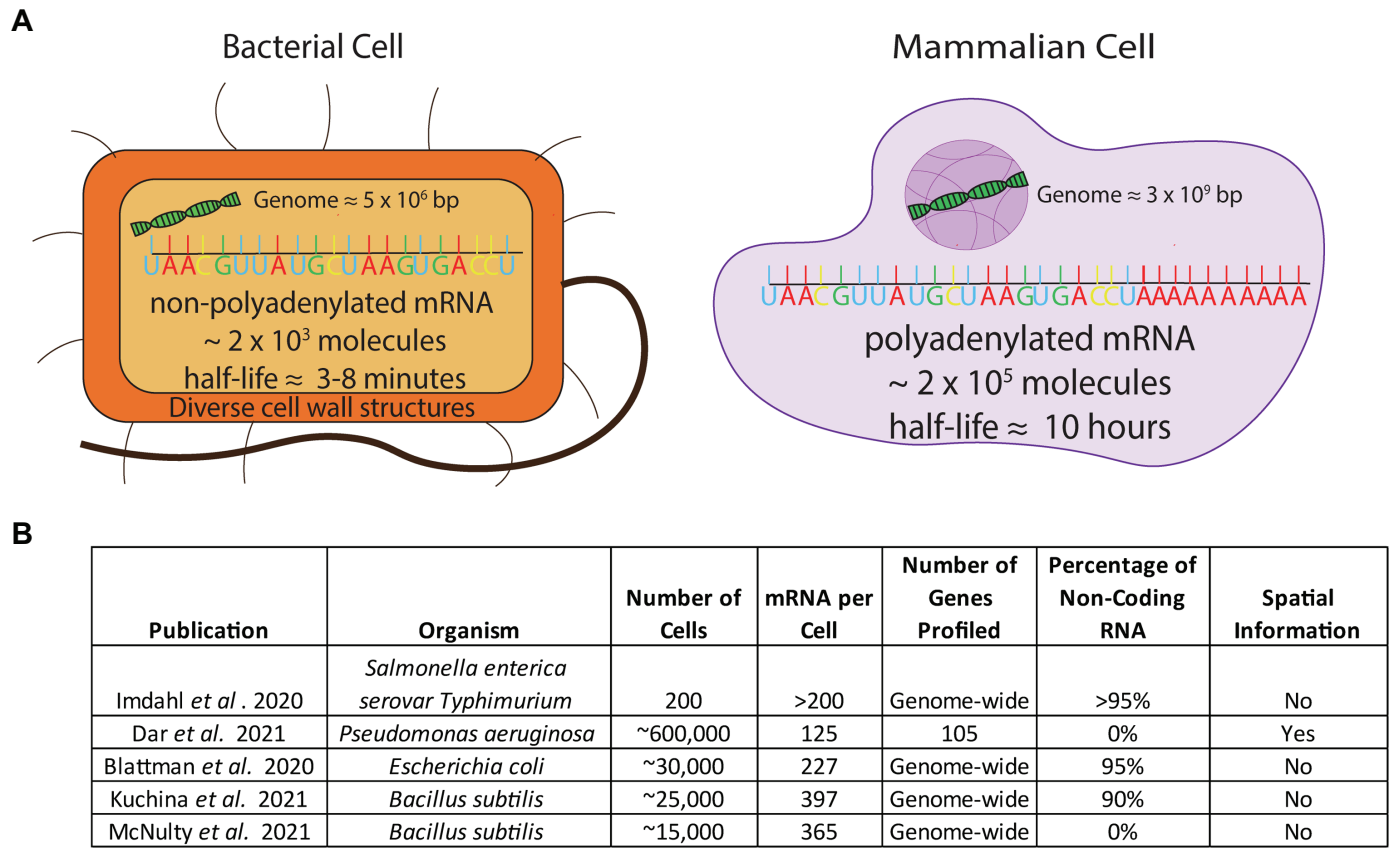
**(A)** Comparison of the challenges in applying scRNA-seq to bacterial cells. Bacterial cells have non-poly-adenylated mRNA transcripts, a low mRNA content, a short half-life, and a high percentage of rRNA composing their transcriptome and have diverse, thick cell walls which make permeabilization difficult **(B)** Studies that have addressed challenges to apply scRNA-seq to bacteria.

Eukaryotic single-cell techniques cannot be directly applied to microbial cells without overcoming several obstacles. Firstly, bacterial mRNA does not contain the 3' poly-adenosine (poly-A) tail that is present in eukaryotic mRNA (Figure 1). The most common eukaryotic techniques use the poly-A tail as a feature to specifically tag mRNA over the much more abundant noncoding RNA (ncRNA) molecules that make up most of the RNA in the cell (>90%). The lack of a consistent poly-A tail on microbial mRNA means that a new method to enrich mRNA would have to be developed. Additionally, bacterial mRNA has a much shorter half-life than mammalian mRNA (minutes versus hours) and is approximately 100-fold less abundant compared to mammalian mRNA (Figure 1; Milo and Phillips, 2015). Addressing this issue would require stabilizing the short lived and sparse mRNA in order for a method to capture the mRNA at a rate high enough to achieve sufficient signal for downstream decomposition/clustering. Lastly, microbial cell walls are diverse and difficult to lyse (Nadal-Ribelles et al., 2019; Blattman et al., 2020; Figure 1) and lysing them require methods that are incompatible with existing mammalian techniques.

Another difference between bacterial and mammalian transcription is the stochasticity of bacterial gene expression (Ackermann, 2015), which is often described as transcriptional bursts and pulses (Chong et al., 2014; Liu et al., 2016). These burst and pulse mechanisms are useful in generating very interesting behaviors (for example see Nadezhdin et al., 2020) but the caveat is that a snapshot of transcriptional activity may not correlate exactly with a stable physiological state in an individual cell in the way that longer lived and more stable eukaryotic signal often does. Therefore, pulsing transcription may be less indicative of a static physiological state of the cell as it is of the activity in a very short and transient moment in a cell’s life. Such transcriptional fluctuation is interesting as a proxy to study gene regulation and can elucidate the mechanisms by which bacteria control gene expression, but there may be times where an abundance of transcripts is not informative of true long-lived physiological states that have meaningful cell–cell interactions within a community. While the influence of fluctuating transcription on genetic regulation is not fully known, there are long-lived cell states in bacteria (Balaban, 2004; Ackermann, 2015). Thus, there is a need to develop experimental and quantitative techniques to discriminate between transcriptional bursts (which are interesting in their own rights) and long-lived states. One way to address this may be to collect samples over a period of time and piece together multiple snapshots of transcription and correlate this data to dynamic measurements in single cells obtained using fluorescent reporter genes and protein markers in timelapse microscopy.

In addition to the challenges of applying scRNA-seq to bacteria, the isolation of a target species from microbial communities can be difficult for microbial ecologists wanting to apply single cell tools to multi-species environments. In multi-species environments, such as the human gut microbiome, some species may be rare. While it is possible to apply single cell tools directly to a mixed population then align reads to an appropriate genome, all current techniques require optimized protocols for each target species due to the variety of cell walls. Another approach for such communities is to isolate bacteria from a mixed community. Bacterial isolation techniques do exist, including fluorescence-activated cell sorting (FACS; Valdivia and Falkow, 1996), and a few reports of magnetic separation based on antibodies (Nakamura et al., 1993) and *in situ* hybridization techniques (Royet et al., 2018). It remains to be seen whether these techniques can enrich sufficient high-quality target cells from a mixed sample for scRNA-seq studies. Taken together, these challenges present the requirement for a highly sensitive and specialized microbial scRNA-seq technique.

### Emerging Bacterial scRNA-seq Techniques

Despite the aforementioned difficulties, in the past year several groups have produced bacterial scRNA-seq datasets of varying size and resolution by utilizing different techniques. One group (Imdahl et al., 2020) used FACS to distribute individual bacterial cells into 96-well plates. The authors were able to characterize heterogeneity when *Salmonella* and *Pseudomonas* were grown on different media; however, due to the technical difficulties and costs associated with FACS-based single cell methods, they only examined 200 cells in total and observed a high background signal from ribosomal RNA (rRNA) reads (97%). In a more high-throughput approach, two other laboratories independently developed similar split-pool indexing techniques to profile the transcriptomes of tens of thousands of bacterial cells (Blattman et al., 2020; Kuchina et al., 2021). These studies – termed PETRI-seq (Blattman et al., 2020) and microSPLiT (Kuchina et al., 2021) couple split-pool barcoding techniques with targeted enrichment of mRNA transcripts by polyadenylation. However, despite this step, noncoding transcripts accounted for over 90% of sequencing reads in both studies. The overall coverage of these techniques ranged from 227 transcripts per cell (Blattman et al., 2020) to 397 transcripts per cell (Kuchina et al., 2021; see Figure 1B). In a third technique for bacterial scRNA-seq, our group introduced a method that overcomes the associated obstacles by coupling DNA probes with a commercial microfluidic droplet generating device (10X Genomics Chromium Controller; McNulty et al., 2021). We generated an average of 5 single-stranded DNA probes per gene spanning the entire genome of organisms of interest, used these probes to hybridize into mRNA, and sequenced the transcripts. We sequenced the transcriptome of over 15,000 individual bacterial cells, detecting approximately 300 transcripts per cell in both *B. subtilis* and *E. coli*. With this method, our group was able to confirm the presence of known bacterial cell states as well as discover previously unknown states. In addition to these single-cell technologies, earlier this year the Newman and Cai laboratories at Caltech successfully generated spatial single-cell bacterial transcriptomics in *Pseudomonas aeruginosa* using a microscopical approach. This dataset probed a large number of cells (∼600,000) but limited the transcript coverage to 105 genes (Dar et al., 2021) as opposed to studies done at the full transcriptome scale. In order to identify distinct cellular states in bacteria with inherently low abundance of transcripts, capturing the highest number of transcripts and number of cells are the two most critical parameters. As new bacterial technologies are developed, the throughput of bacterial scRNA-seq data will continue to improve.

### Investigating Microbial Heterogeneity

**Figure 2:**
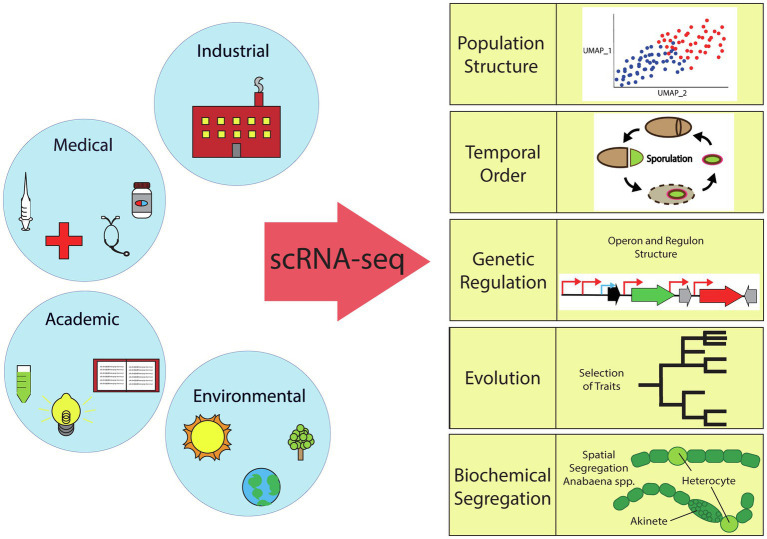
Overview of the scientific fields impacted by bacterial single-cell RNA sequencing. scRNA-seq can elucidate bacterial population structure (TSNE or UMAP plots), temporal order (sporulation cycle, etc.), genetic regulation (activation/repression), evolution (selection of traits), or biochemical segregation (spatial or temporal).

Bacterial scRNA-seq can address many long-standing questions of basic understanding in microbial systems of particular importance to the industrial, medical, and environmental fields. As outlined in this section and depicted in Figure 2, the simplest level of information provided by bacterial scRNA-seq is the basic characterization of the inherent heterogeneity (population structure) in different species and growth conditions. A more detailed analysis focusing on aligning cells based on pseudotime or real temporal measurements can reveal temporal organization and the ordering of events in the context of cellular differentiation. Additionally, single-cell data can be used to tease out the mechanisms by which this differentiation is regulated. In the context of laboratory-based evolution studies, the evolution of social interactions and the relationship between evolved genotype and transcriptional phenotype can be compared. Finally, our ultimate hope is that scRNA-seq will pave the way for understanding the fundamental forces that drive and organize heterogeneity.

#### Single-Cell RNA-seq Allows High-Throughput Characterization of Population Structure

The ability of scRNA-seq to rapidly and affordably profile the transcriptional signature of thousands of cells has been used in mammalian systems to characterize the presence of different cell types in laboratory cultured cells, tissues, and whole organisms (Klein et al., 2015; Wagner et al., 2018; Kimmel et al., 2019). These studies have provided inventories of cells present (cell atlases; Hao et al., 2020; The Tabula Muris Consortium, 2020) and documented changes across cells from different conditions. This characterization can lead to the discovery of rare cell types, such as antigen-specific B and T cells (Nguyen et al., 2018) and continues to identify previously unknown cellular states even in well-studied systems (Hildreth et al., 2021).

In bacterial systems, the first reports were able to provide this information and the structure and presence of known populations, such as sporulation and competence, have been reported (Kuchina et al., 2021; McNulty et al., 2021). Similarly, a rare subpopulation (<1%) was discovered in *Bacillus subtilis* expressing PBSX prophage genes (Kuchina et al., 2021), a rare prophage-induced subpopulation of *S. aureus* (Blattman et al., 2020), and a new cell type expressing arginine synthesis genes was discovered in *Bacillus subtilis* (McNulty et al., 2021).

#### Single-Cell RNA-seq Reveals Temporal Events

Single cells can be ordered in 2D space according to stages of differentiation or cell states, and the advancement through this order represents a pseudotime progression (Trapnell et al., 2014; Rosenberg et al., 2018). For example, developmental studies show the transcriptional progression of different cell types in a fetus over time (He et al., 2020). Cancer cells gain mutations over time as the cancer develops, and these developmental trajectories can be delineated both using pseudotime analysis (Caron et al., 2020) as well as comparing across longitudinal (serial) biopsy samples from the same donor (Guruprasad et al., 2020). In aging studies, an increase in senescent cell populations and the development of cells with distinct phenotypes, such as blood cells that appear to be pre-neoplastic (pre-cancerous), have been reported (Kimmel et al., 2019; The Tabula Muris Consortium, 2020).

In bacterial systems, there are clues that such temporal ordering can be studied, though no analysis has focused on this aspect thus far. scRNA-seq has demonstrated changes over time in bacterial gene expression and suggests that some cellular processes may be more prone to heterogeneity than others (Blattman et al., 2020; Kuchina et al., 2021; McNulty et al., 2021). One study followed scRNA-seq data through growth of *B. subtilis* in rich media and observed gene expression profiles associated with competence and prophage induction. While they identified a novel gene expression state in a subpopulation with activation of a niche metabolic pathway (myo-inositol catabolism) only in the later OD time points, a deeper analysis of that subpopulation was not discussed (Kuchina et al., 2021). In another study, we observed upregulation of multiple known early sporulation factors (sigG, spooA, and spoIIAB) in a metabolically active subpopulation of *B. subtilis* grown in minimal media (McNulty et al., 2021). This subpopulation of cells was in stark contrast to other cells within the metabolically active population and may indicate an early commitment to sporulation.

#### Gene Regulatory Cascades Are Identified by Single-Cell RNA-seq

Using time course and pseudotime studies, it is possible to uncover the order in which different regulatory features are expressed therefore providing insight about the mechanisms of development. When pseudotime algorithms, such as Monocle, Slingshot, P-Creode (Trapnell et al., 2014; Herring et al., 2018; Street et al., 2018), or others, informatically order the progression of cells through differentiation, novel factors can be discovered with a projection of the cell’s trajectory. Specifically, in the original study that introduced Monocle, eight previously undiscovered transcription factors that influenced the course of myoblast (precursors to muscle cells) differentiation were identified in a well-studied regulation system (Trapnell et al., 2014). These new discoveries demonstrated the ability of scRNA-seq to uncover previously missed transcriptional regulators. There are no bacterial studies using pseudotime analysis to date, but we envision that these tools will be used extensively for bacterial scRNA-seq analysis. In addition to these pseudotime tools, measurements of RNA velocity (La Manno et al., 2018) can also inform the dynamics of gene expression. However, RNA velocity measurements (La Manno et al., 2018) rely on analysis of processed RNA (splicing) and would need to be adapted for bacterial use.

#### Single Cell RNA-Seq Ties Genotype and Phenotype Together in Lab-Based Evolution Studies and Provides Insight Into the Underlying Forces That Govern Cellular Heterogeneity

Experimental evolution studies have uncovered many rules of natural selection in many organisms (Kawecki et al., 2012). Experimental evolution studies using microbes have made many contributions to better understand evolutionary processes because of their fast generation time and small genomes, coupling quick adaptation with straightforward genetic analysis.

In several systems, it has been demonstrated that a fraction of the cellular population may dynamically switch their phenotype when subject to changing environments. Such switching involves cells entering and exiting physiological states in which specific gene sets are expressed and have been shown to have benefits for the community in some instances (Ackermann, 2015; Rosenthal et al., 2018). The existence of phenotypic cell switching may be advantageous for adaptation because it allows the population to deal with stressful environmental conditions until cells acquire mutations that are best suited for the particular stress. The control of such phenotypic cell switching dynamics should thus be traits that are under genetic selection under certain conditions. State switching is more common under fluctuating environments compared to static environments, but the control of switching dynamics has not been deeply studied due to the difficulties of obtaining genome-wide measurements of heterogeneity in single cells (Murren et al., 2015). With bacterial scRNA-seq evolutionary studies, we will be able to identify physiological states, and the level of phenotypic switching can be measured by timelapse microscopy to determine the conditions that give rise to these phenomena. These learnings can potentially inform what evolutionary forces drive genetically clonal cells to specialize into different cell types – as seen in multicellular life forms, a fundamental question and can provide insight into the origins of multicellularity and its drivers.

In some cases, it has been demonstrated that cellular processes must segregate into either distinct cells, locations, or specific times in order to avoid the deleterious effects of having metabolically incompatible processes co-occur in a single cell. For example, oxygen radicals are known to destabilize nitrogenase, so aerobic respiration is sequestered from nitrogen fixation in microorganisms (Haselkorn, 1978). In the case of the cyanobacteria *Anabaena*, this sequestration is achieved by differentiation of a cell fraction into a specialized nitrogen-fixing cell type (heterocyst; see Figure 2).

In addition to growth in suspension, microenvironments may drive cellular differentiation through the accumulation and depletion of nutrients and byproducts. While the specific microenvironments that effect different microbes vary greatly, as well as the evolutionary strategies developed by microbes to address environmental obstacles that arise inherently with growth, it is interesting to speculate that there are some strategies that recur throughout biology.

Microbes are exposed to high environmental variability, raising the question whether distinct cell types are truly conserved throughout the bacterial world? Our hypothesis is that the space for bacterial heterogeneity across the entirety of microbiology is vast; however, we posit that even within this space there are rules that govern which processes segregate and that these rules can be identified via high-throughput characterization of single cells that compose communities.

## Data Availability Statement

The original contributions presented in the study are included in the article/supplementary material, further inquiries can be directed to the corresponding author.

## Author Contributions

MB and AR came up with the idea for this perspective and wrote the manuscript. All authors contributed to the article and approved the submitted version.

### Conflict of Interest

The authors declare that the research was conducted in the absence of any commercial or financial relationships that could be construed as a potential conflict of interest.
